# A loss of function variant in CASP7 protects against Alzheimer’s disease in homozygous APOE ε4 allele carriers

**DOI:** 10.1186/s12864-016-2725-z

**Published:** 2016-06-23

**Authors:** Kristin L. Ayers, Uyenlinh L. Mirshahi, Amr H. Wardeh, Michael F. Murray, Ke Hao, Benjamin S. Glicksberg, Shuyu Li, David J. Carey, Rong Chen

**Affiliations:** Department of Genetics and Genomic Sciences, Icahn Institute for Genomics and Multiscale Biology, Icahn School of Medicine at Mount Sinai, New York, NY 10029 USA; Geisinger Clinic, Danville, PA 17822 USA

**Keywords:** Alzheimer’s disease, Genetic variants, Protective alleles, CASP7, Resilience, Loss of function

## Abstract

**Background:**

Alzheimer’s disease (AD) represents the most common form of dementia in elder populations with approximately 30 million cases worldwide. Genome wide genotyping and sequencing studies have identified many genetic variants associated with late-onset Alzheimer’s disease (LOAD). While most of these variants are associated with increased risk of developing LOAD, only limited number of reports focused on variants that are protective against the disease.

**Methods:**

Here we applied a novel approach to uncover protective alleles against AD by analyzing genetic and phenotypic data in Mount Sinai Biobank and Electronic Medical Record (EMR) databases.

**Results:**

We discovered a likely loss-of-function small deletion variant in the caspase 7 (CASP7) gene associated with significantly reduced incidence of LOAD in carriers of the high-risk APOE ε4 allele. Further investigation of four independent cohorts of European ancestry revealed the protective effect of the CASP7 variant against AD is most significant in homozygous APOE ε4 allele carriers. Meta analysis of multiple datasets shows overall odds ratio = 0.45 (*p* = 0.004). Analysis of RNA sequencing derived gene expression data indicated the variant correlates with reduced caspase 7 expression in multiple brain tissues we examined.

**Conclusions:**

Taken together, these results are consistent with the notion that caspase 7 plays a key role in microglial activation driving neuro-degeneration during AD pathogenesis, and may explain the underlying genetic mechanisms that anti-inflammatory interventions in AD show greater benefit in APOE ε4 carriers than non-carriers. Our findings inform potential novel therapeutic opportunities for AD and warrant further investigations.

**Electronic supplementary material:**

The online version of this article (doi:10.1186/s12864-016-2725-z) contains supplementary material, which is available to authorized users.

## Background

The prevalence of dementia in the Western population over age of 60 has been estimated to exceed 5 %. Alzheimer’s disease (AD) is the leading cause of dementia accounting for approximately 30 million cases worldwide [1, 2]. The incidence and prevalence of AD is age dependent, doubling every 5 years after age 65 [3]. As the proportion of population over 60 years of age continues to rise, the number of individuals developing AD will also increase, presenting a major health issue worldwide. Pathologically, AD is defined by extensive neuronal loss and accumulation of extracellular amyloid plaques formed by aggregation of β-amyloid (Aβ) peptides and intracellular neurofibrillary tangles composed of hyper-phosphorylated tau proteins [4].

AD has a strong genetic component. It has been generally recognized that there are two major categories of AD, familial early-onset AD (EOAD) and sporadic late-onset AD (LOAD). EOAD represents less than 1 % of all AD cases and they are characterized by early-onset, often before age 60, and Mendelian inheritance occurring mostly in an autosomal dominant manner. Linkage analysis of EOAD families in the 80’s and early 90’s led to the discoveries of fully penetrant disease causing mutations in *APP* encoding β-amyloid precursor protein, *PSEN1* and *PSEN2* encoding components of the γ-secretase complex, presenilin 1 and presenilin 2 respectively [5]. In amyloidogenic pathway, APP, a transmembrane protein is first cleaved by a β-secretase, encoded by *BACE1* and subsequently by the γ-secretase complex to form Aβ peptides [6, 7]. Identification of disease causing mutations in *APP*, *PSEN1*, *PSEN2* underscores the pathogenic role of the amyloidogenic pathway in AD development [8, 9].

Linkage studies, genome-wide association studies (GWAS), and recent whole exome sequencing (WES) have identified dozens of risk genes in LOAD [4, 5, 10]. These risk genes have created a broader picture of pathways involved in AD pathogenesis. Several pathways have been highlighted by these genes, including cholesterol metabolism (APOE, CLU, ABCA7), immune response (CR1, CD33, MS4A, TREM2) and endocytosis (BIN1, PICALM, CD2AP, EPHA1, SORL1) [4]. Among AD risk genes and variants, APOE is the strongest risk factor. APOE has three isoforms determined by cysteine-to-arginine substitutions at amino acid position 112 and 158, corresponding to two SNPs (rs429358 and rs7412 respectively) [11, 12]. The three isoforms are referred as APOE2 (cys112, cys158), APOE3 (cys112, arg158) and APOE4 (arg158, arg158) with the corresponding alleles designated ε2, ε3, and ε4 respectively [13]. APOE ε3 is the most common isoform with 60-70 % [14] allele frequency. APOE ε4 allele is associated with increased AD risk in both familial EOAD and sporadic LOAD, with 2–5 fold increased risk for heterozygous carriers and 12–15 fold increased risk for homozygous carriers in Caucasian populations [5, 15]. These risks have been estimated for as a 5.7 fold increase in homozygous and no increased risk in heterozygotes in the African American population [16]. In Hispanics, this risk is estimated to be 2.2 fold in homozygotes with no increased risk in heterozygotes [16]. However, there is a greater prevalence of Alzheimer’s and other dementias in African-Americans and Hispanics, suggesting other environmental or genetic factors are at play [17].

Elucidating the functional effects of naturally occurring genetic variants is one of the major challenges in genetic studies of human diseases [18]. With most of the genetic studies focused on variants associated with increased AD risks, there are a limited number of reports discussing variants that render protective effects against AD. The most notable example is the APP A673T mutation protecting against AD as well as cognitive decline in the elderly without AD due to 40 % reduction in the formation of Aβ peptides [19]. Missense variants in several other genes associated with lowered risk of AD or neuronal atrophy, including TREML2 [20], HMGCR [21], and REST [22], have recently been described. In this study, we applied a novel approach to discover AD protective variants by identifying genetic modifiers for AD risk in APOE high-risk ε4 allele carriers. Genotyping data of approximately one million markers plus 37 million imputed SNPs in Mount Sinai Biobank [23] were analyzed and a small deletion variant (rs10553596) in the coding region of caspase 7 gene (CASP7) was found to be significantly associated with reduced incidence of AD and dementia in APOE ε4 carriers. The protective effect of rs10553596 is observed in four independent LOAD cohorts. Interestingly, the protective effect of this CASP7 variant appears to be most significant in homozygous APOE ε4 carriers. At gene expression level, eQTL analysis indicated that the rs10553596 variant is correlated with lowered caspase 7 expression. These results provide new insights into the underlying genetic mechanism of AD as well as opportunities for novel therapeutic strategies.

## Methods

### Study participants or study cohorts

We analyzed 6 datasets (Table 1): Mount Sinai Biobank (~14 K individuals with genotype data), Geisinger Health System (GHS) MyCode Cohort (9856 unrelated individuals, [24]), GBAD (1588 individuals, http://www.ncbi.nlm.nih.gov/projects/gap/cgi-bin/study.cgi?study_id=phs000219.v1.p1), ADNI (2826 individuals, http://adni.loni.usc.edu), ADSP (10,939 individuals, https://www.niagads.org/adsp/content/home), and ADGC (6065 individuals, http://www.ncbi.nlm.nih.gov/projects/gap/cgi-bin/study.cgi?study_id=phs000372.v1.p1). ADSP is a large AD cohort including whole genome sequencing data. However, after downloading all APOE ε4 homozygotes and filtering for a read count ≥10 for the CASP7 variant, only one control remained (Table 1), and thus the data was not included in the meta-analysis.

**Table 1:** The analyzed datasets in this study

Dataset	Samples	Cases	Controls	APOE ε4 Homozygotes….		
				All	Cases	Controls
Mt. Sinai All	13,710	238	5325	93	8	85
White				*25*	*1*	*24*
African American				*37*	*3*	*34*
Hispanic/Latino				*28*	*4*	*24*
GHS	7645	287	7358	132	6	126
GBAD	1588	806	782	103	92	11
ADNI	2826	370	569	91	55	28
ADGC	6065	3666	1604	456	419	25
ADSP	10,939	6311	4628	145	96	1
TOTAL	42,773	12,248	20,067	1020	572 (EUR > 60)	190 (EUR > 60)

Some of these numbers may not sum due to various filtering steps. The final case/control totals for the APOE ε4 homozygotes include only those analyzed in the meta-analysis, and thus do not include the Mt. Sinai or ADSP datasets

### The Mount Sinai Biobank cohort

Mount Sinai Biobank was established in 2007 in New York city and is distinguished among most biobanks because the participants are recruited from highly diverse communities, with the majority being either Hispanic or African American [23]. Currently it has collected DNA samples from more than 30,000 enrolled participants, and genotyping data has been generated for more than 14,000 patient samples using Illumina OmniExpressExome-8 v1.1 BeadChip that covers approximately one million genetic markers. Available clinical information for all the participants such as disease diagnosis, laboratory test results and medication history were obtained from Mount Sinai electronic medical record (EMR) databases.

### Genotyping and imputation

SNP genotypes were obtained using Illumina OmniExpressExome-8 v1.1 BeadChip (Mt. Sinai), Illumina Infinium ExomeCore 12v1.0_B (GHS), Affymetrix 500 K array set (GBAD), Illumina Human610-Quad BeadChip and Illumina HumanOmniExpress BeadChip (https://www.synapse.org/#!Synapse:syn2290704/wiki/64710) (ADNI), and Illumina Human660W-Quad/HumanOmniExpress (ADGC).

Quality control was performed on all genotype datasets prior to imputation. In the Mount Sinai Biobank, individuals with discordant sex, call rates below 98 %, or out-lying heterozygosity were removed. SNPs with call rates below 95 % or with deviation from Hardy-Weinberg equilibrium (HWE) with *p*-value < 5e-5 were also excluded. For the GHS dataset, genotypes were pre-cleaned and imputed separately as previously described [25]. Samples with sex mismatches and low call rates and SNPs with low call rates were removed. For the GBAD Affymetrix 500 K dataset, the data was pre-QCed according to the protocol given with the data [26]. The GBAD data samples with call rates <94 % or gender mismatches were removed along with SNPs with HWE *p*-value < 5e-7 or call rate <95 % (99 % for MAF <0.05). For the ADNI dataset, samples with call rates <98 %, population outliers, and relateds were removed along with SNPs with HWE *p*-value < 0.001, minor allele frequency (MAF) < 0.05, or call rate <98 % according the protocol at http://adni.loni.usc.edu/ and https://www.synapse.org/#!Synapse:syn2290704/wiki/64710. For the ADGC dataset, data samples with call rates <99 %, gender mismatches, and individuals with outlying heterozygosity were removed along with SNPs with HWE *p*-value < 1e-10, call rate <98 %, and MAF < 1 %.

All genotype datasets were prephased using SHAPEITv2 [27] and imputation was performed with IMPUTE2 [28] using the 1000 genomes integrated variant set as the reference panel. The Mount Sinai Biobank, the GHS dataset, the ADGC dataset, and the GBAD dataset were imputed with the phase III haplotype reference panel, while the ADNI dataset was pre-imputed with the phase I panel. The imputation info score for the CASP7 variant rs10553596 was 0.97 in the Mount Sinai Biobank, and 0.99 in the GBAD, ADGC and GHS cohorts. For the GHS, GBAD, ADGC, and ADNI datasets, individuals who had a posterior probability of less than 0.9 for the most likely genotype were excluded from the analysis. The CASP7 variant rs10553596 was imputed in all datasets.

### APOE ε4 status

Individuals who have the C/C genotype at rs429358 are obligate homozygous ε4, and those who harbor the T/T genotype as rs7412 are obligate non-ε4-carriers. Individuals who were C/C at rs429358 and T/T at rs7412 were removed from the analysis, as this haplotype is not observed (and it is thus due to genotype error or incorrect genotype imputation). In the Mt. Sinai Biobank, rs7412 was genotyped while rs429358 was imputed with imputation info of 0.96. Two individuals were excluded from the analysis due to an inconsistent haplotype. In the GHS cohort, 2033 individuals were identified as APOE ε4 carriers (rs7412 was genotyped and rs429358 was imputed), of which 812 carry at least 1 allele for CASP7 rs10553596. In the GBAD dataset, rs429358 and rs7412 were genotyped independently. APOE ε4 status and the CASP7 rs10553596 genotype could be estimated for 1423 of the 1588 individuals. The ADNI dataset included the number of copies of the APOE ε4 allele (both rs7412 and rs429358 were genotyped). There were 919 individuals of European descent with APOE ε4 status. The ADGC dataset provided the two APOE alleles for each individual.

### Phenotype determination

Since EOAD occurs in individuals before the age of 60, we selected individuals older than 60 in all cohorts as one of the criteria to define LOAD. For both the Mount Sinai Biobank and GHS EMR data, AD cases were selected using ICD9 billing codes 290, 294, and 331 and all their subtypes (excluding Reye’s syndrome (331.81) and alcohol induced dementia (291.2)) from electronic medical records (EMR). Cases were defined as individuals with first record of AD/dementia diagnoses above age 60. Controls were those over 60 year of age at their last recorded visit with no records including ICD9 290, 294, and 331. In the GBAD dataset, LOAD cases and controls were defined according to the protocol at http://www.ncbi.nlm.nih.gov/projects/gap/cgi-bin/study.cgi?study_id=phs000219.v1.p1. For the ADNI dataset, visits were recorded for patients in 3 categories: NL (normal), MCI (mild cognitive impairment), or Dementia. For these individuals, we used the most severe diagnosis recorded. Thus, individuals recorded as NL and MCI were considered controls, and those recorded as Dementia were considered cases. Only individuals recorded as ‘White’ and ‘Not Hispanic/Latino’ and who were over the age of 60 were analyzed. The ADGC dataset includes individuals with normal phenotype, AD, or MCI. Individuals classed as normal/MCI who were over 60 years of age at the last visit were considered controls, and AD patients with initial diagnosis after age 60 were considered cases. Only individuals recorded as “White” and “Not Hispanic/Latino” who clustered together in PC analysis were analyzed.

### Meta analysis

A weighted fixed-effect meta-analysis was performed in the R package metafor assuming a dominant model for the CASP7 rs10553596 variant in homozygous APOE ε4 carriers, heterozygous ε4 carriers, and non-carriers (adding a half count to any zero count cells). To test for any possible population stratification issues within the European cohorts, we included the first 3 principle components (PCs) as covariates in a logistic regression model for the APOE ε4 homozygotes for each of the ADNI, ADGC, and GBAD datasets. The PCs were not statistically significant nor did they have a notable impact on the estimated odds ratio.

## Results and discussion

### Identification of genetic variants associated with reduced AD prevalence in APOE ε4 carriers

We first performed an exploratory analysis in the Mount Sinai Biobank to find protective alleles for APOE ε4 homozygotes, who are at high risk for AD. The SNP rs429358, used to determine APOE ε4 status, is well known for being difficult to genotype and failed QC on our array. The imputed genotypes were used to estimate the APOE ε4 status. Though this variant has overall high imputation quality, genotype imputation is more difficult in populations of African ancestry, due to lower linkage disequilibrium (LD) and higher genetic diversity. If we were to use a cutoff of genotype posterior probability > 0.9 for the imputed rs429358 SNPs, this would result in only 29 individuals above age of 60 being assigned as homozygous APOE ε4 carriers, of which only 2 were diagnosed with AD/dementia. We decided to use a loose threshold of genotype posterior probability (>0.5) for the APOE SNP rs429358 in order to increase sample size for a more powerful test of significance. We applied this threshold to all tested imputed variants. Using ICD9 billing codes for AD and dementia diagnosis (290, 294 and 331), we had 8 AD/dementia patients and 72 controls. With a one-sided Fisher’s exact test, we found 12 annotated loss-of-function variants with *p*-value <0.05 that were protective for AD/dementia. The top two variants were in SLC22A24 (a putative ion transporter with unknown functional relevance to AD) and MUC19 (a gene known to give false positive associations due to its size). The third most significant variant was in CASP7, rs10553596, with an odds ratio of 0.080 and *p*-value of 0.0062. In this variant, two nucleotides (TT) is deleted in CASP7 coding region resulted in leucine to serine change at amino acid position 44 (mRNA accession: NM_001267057.1; protein accession: NP_001253986.1). In addition, the TT deletion causes a premature termination codon downstream at amino acid position 133. Therefore, it is highly likely this is a loss-of-function variant. The association of this variant to AD in the APOE ε4 heterozygotes (odds ratio = 0.75, *p*-value = 0.37) suggests that the protective effect of rs10553596 against AD in all APOE ε4 carriers might be present mainly in the APOE ε4 homozygous population. Rs10553596 is a relatively common variant in general population, with differing allele frequencies amongst the different 1000 genomes populations (Additional file 1: Figure S1).

Next, we reinvestigated rs10553596 with a more stringent threshold for the imputed variants to determine if an association still holds. To increase the size of the test group, we performed a case-control analysis of genotypes between those diagnosed with AD or dementia and those without among APOE ε4 carriers. Using the combined posterior probability of homozygous and heterozygous rs429358 and the information from the other genotyped APOE SNP rs7412, we can be fairly confident whether or not an individual carries at least one copy of APOE ε4 (we can apply the same rational to the imputed variant CASP7 variant rs10553596 since we are analyzing a dominant model). With a cutoff for the combined posterior probability > 0.8, 842 individuals above age of 60 and in the top three largest ethnic groups were identified as APOE ε4 carriers (Table 2a). As the control group, 3666 were identified as non-APOE ε4 carriers and are above age of 60 (Table 2b). 50 of the 842 APOE ε4 carriers, and 157 of the 3666 individuals in the control group were diagnosed with AD or dementia. This represents an odds ratio of 1.4 fold for AD risk in APOE ε4 carriers. The result shows that presence of at least one copy of the rs10553596 alternate allele significantly lowers the AD risk (odds ratio = 0.56, p = 0.031) in APOE ε4 carriers (Table 2a). However, when the same analysis was performed on non-APOE ε4 carriers, no significant association between rs10553596 and reduced AD risk was detected (Table 2b) although a trend was observed. This suggests that rs10553596 is not protective for AD by its own, but is most likely a modifier for APOE ε4 alleles on AD risk.

**Table 2:** Association between CASP7 rs10553596 and AD/dementia status in APOE ε4 allele carriers (A), and non-APOEε 4 allele carriers (B), stratified by ethnicity in Mount Sinai Biobank

A. Carriers: Overall *P*-value = 0.031, OR = 0.56 (95 % CI 0.00–0.94)					
Ethnicity	Phenotype	Ref-Ref	Alt-*	*P*-value	Odds Ratio
European	Controls	137	141	0.25	0.49
	AD/dementia	6	3		
African	Controls	68	145	0.19	0.54
	AD/dementia	7	8		
Hispanic	Controls	155	146	0.12	0.56
	AD/dementia	17	9		
B. Non-Carriers: Overall *P*-value = 0.060, OR = 0.77 (95 % CI 0.00–1.01)					
Ethnicity	Phenotype	Ref-Ref	Alt-*	*P*-value	Odds Ratio
European	Controls	588	637	0.43	0.83
	AD/dementia	10	9		
African	Control	233	453	0.24	0.74
	AD/dementia	14	20		
Hispanic	Control	867	731	0.17	0.80
	AD/dementia	62	42		

### Evaluation of rs10553596 as an AD protective variant

CASP7 encodes a member of the cysteine-aspartic acid protease (caspase) family. Sequential activation of caspases plays a central role in the execution-phase of cell apoptosis. However, the function of caspase 7 is beyond its role in apoptosis. For example, it has been illustrated that microglia cell activation by inflammatory signals are mediated through caspase cascade involving caspase 3 and caspase 7, without triggering apoptosis [29]. Microglial are resident immune cells in brain acting as the main form of immune response in central nervous system (CNS). Abnormal microglial activation causes neurotoxicity and plays a predominant role in both AD and Parkinson’s disease (PD) pathogenesis. A recent study has shown that a LD haplotype block containing the CASP7 gene is associated with reduced AD risk in Caribbean Hispanic populations [30].

**Fig. 1:**
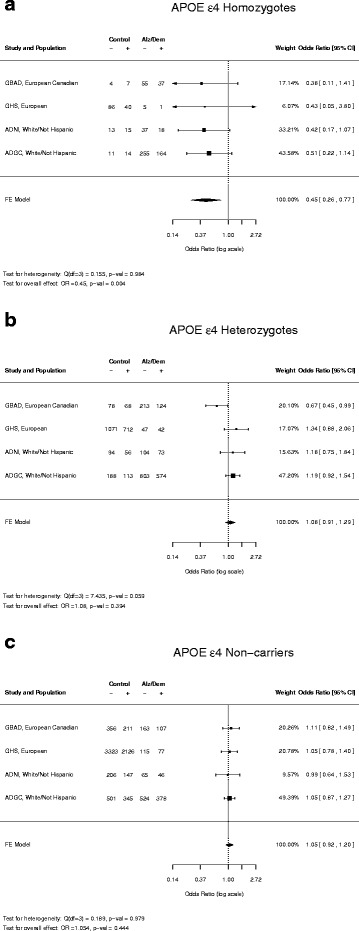
Meta-analysis of association between CASP7 rs10553596 and AD prevalence in homozygous APOE ε4 carriers (**a**), heterozygous APOE ε4 carriers (**b**) and non-carriers (**c**). Control: individuals not diagnosed with AD or dementia. Alz/Dem: individuals diagnosed with AD or dementia. “−” and “+” denote individuals with both ref alleles of rs10553596, or harboring at least one alt allele of rs10553596, respectively

We then attempted to replicate our findings in five independent cohorts (Table 1). (1) The GBAD study [26, 31], with French Canadian ancestry, (2) the ADNI study [32], (3) the GHS MyCode study [24], (4) the ADGC study [33], and (5) the ADSP study (http://www.ncbi.nlm.nih.gov/projects/gap/cgi-bin/study.cgi?study_id=phs000572.v1.p1). We assigned each individual into one of the following three populations: APOE ε4 homozygotes, APOE ε4 heterozygotes and non-APOE ε4 carriers based on their APOE genotypes. For the ADSP WGS dataset, after genotype calling, we were left with only one APOE ε4 homozygote control, and thus the data was not included in the meta-analysis. Because these cohorts are of primarily of European ancestry, one can achieve higher accuracy of genotype imputation due to longer stretches of LD and lower genetic diversity. The protective effect of CASP7 variant rs10553596 was then assessed for individuals with genotype posterior probability of >0.9 for any genotype in each of the three sub-populations (APOE ε4 homozygotes, APOE ε4 heterozygotes and non-APOE ε4 carriers). The combined meta-analysis *p*-value (odds ratio) for these four cohorts was 0.004 (0.45) in homozygous APOE ε4 carriers (Fig. 1a), indicating a significant protective effect of CASP7 rs1055359. In contrast, this protective effect of the rs10553596 variant is not observed in APOE ε4 heterozygotes (Fig. 1b) and non-carriers (Fig. 1c).

Interestingly, when we interrogated a centralized genetic variant repository (DIVAS) we built that included genotyping and sequencing data of 150,000 individuals from multiple disease cohorts [34], we observed that allele frequency of rs10553596 is significantly lower in familial Amyotrophic lateral sclerosis (ALS) at 5 % in comparison to 19–50 % in healthy populations (Additional file 1: Figure S2), implicating a protective effect of this variant against ALS, another neuro-degenerative disease. The familiar ALS cohort was downloaded from dbGap with accession phs000101.v4.p1 [35, 36]. Although this observation requires further validation given rs10553596 is an indel variant and the technical challenges associated with indel calling, it is consistent with our findings on the protective effect of rs10553596 in LOAD. We further assessed the effect of the rs10553596 variant on CASP7 gene expression through eQTL analysis. As demonstrated in Table 3, the alternate allele of rs10553596 is significantly associated with lowered CASP7 expression in the tissues we examined, including several brain tissues such as prefrontal cortex, visual cortex and cerebellum. This supports that rs10553596 is a loss of function variant for CASP7.

**Table 3:** rs10553596-c allele is associated with lower CASP7 gene expression level

Tissue	Reporter ID	Effective allele	eQTL *P*-value	eQTL direction	Sample size
Lung	100139172_TGI_at	c	2.6e-44	↓	1111
Prefrontal cortex	10023817180	c	1.5e-21	↓	583
Visual cortex	10023817180	c	4.0e-11	↓	409
Cerebellum	10023817180	c	5.7e-12	↓	496
Liver	10023817180	c	2.1e-23	↓	563
Omental fat	10023817180	c	2.6e-62	↓	675
Subcutaneous fat	10023817180	c	1.6e-29	↓	611

Expressional QTLs (eQTLs) between rs10553596 and CASP7 expression level were reported in large tissue collections, including lung [40], brain regions [41], liver [42] and fat tissues [42]. In lung eQTL study, Affymetrix HU133 array was used; and in other eQTL studies, Agilent Human44K1.1 array were employed

African American and Hispanic APOE ε4 carriers have a lower risk of developing Alzheimer’s or dementia than European carriers. The allele frequencies of the CASP7 variant rs10553596 differ among the 1000 genomes ethnicities: 34–48 % in African populations, 21–32 % in South Asian populations, 19–29 % in European populations, 21–28 % in East Asian populations, and 15–28 % in American populations (Additional file 1: Figure S1). The higher allele frequency of rs10553596 in African populations might explain why APOE ε4 African Americans have a lower risk for Alzheimer’s than Europeans [17]. It is identified in this study, possibly due to the unique composition of population in Mount Sinai Biobank with the majority being either African American or Hispanic [23], although we were able to confirm the protective effect of rs10553596 against AD in four European cohorts. If we plot the frequency of rs10553596 versus the estimated APOE ε4 frequencies, we see a general positive correlation (*r*^2^ = 0.42), which may reflect selective pressure (Additional file 1: Figure S1). We also realize that diversity of the frequency of this marker and the varying risks amongst ethnicities may inflate association due to subtle population stratification issues. While this is definitely a possibility in the Mount Sinai Biobank due to complex admixtures, it is far less likely to be a major factor in the more homogenous cohorts of European descent (principle component analysis supports this conclusion, see Methods).

The protective effect of rs10553596 against LOAD is only observed in APOE ε4 homozygote populations (Fig. 1). It has been well documented that APOE binds to Aβ and influences the clearance of soluble Aβ and the Aβ aggregation [4]. However it was unclear whether APOE genotype affects Aβ-plaque-associated neuro-inflammation until a recent study demonstrated that in addition to altering morphological profiles of Aβ deposition, APOE genotype influences Aβ-induced glial activation in cortex [37], a key step leading to neuro-inflammation in AD pathogenesis. Given the critical role of caspase 7 in microglial activation and recent findings implicating APOE ε4 alleles in modulating neuro-inflammatory responses in AD development, it is only fitting that the loss-of-function alt allele of rs10553596 in CASP7 exhibited protective effect against AD in APOE high-risk ε4 carrier populations. Moreover, the protective effect of a CASP7 loss-of-function variant against AD only in APOE ε4 homozygotes may explain the clinical observation that anti-inflammatory interventions in AD show greater benefit in APOE ε4 carriers as opposed to non-carriers [38]. As pan-caspase inhibitors have been under development for several autoimmune diseases [39], our discovery has potentially significant clinical relevance implicating novel therapeutic opportunities for AD.

We also recognize the limitations in our study. Limited sample size is an inherent challenge. Two of the 6 datasets, Mount Sinai Biobank and Geisinger Health System (GHS) MyCode Cohort, are population-based data that is not focused on AD. Therefore, the frequency of APOE ε4 homozygous carriers is equal to the population frequency of approximately 1-2 %. Multiple filtering steps throughout the analyses (see Methods) further reduced the sample size. In addition, there are a significantly smaller number of AD patients than controls. In contrast, the 4 AD datasets (GBAD, ADNI, ADGC and ADSP) have significantly larger number of AD patients than controls among APOE ε4 homozygous carriers since the APOE ε4 allele is enriched in AD populations. Unbalanced AD vs. control sample size poses a great challenge to achieve the level of statistical significance reported in typical GWAS studies with balanced case vs. control populations of thousands to tens of thousands in sample size. Even with an original sample size of nearly 40,000 individuals, we were left with <1000 homozygous APOE ε4 individuals for the meta-analysis (Table 1; Fig. 1). Another limitation is that we define age 60, a commonly used threshold as a cutoff for LOAD. It is likely that some of the individuals in the control population will develop AD as they age. Therefore, our analyses suggest that the CASP7 variant rs10553596 protects against AD or simply delays the age of onset. As more AD genotyping and genomic sequencing data from large consortium become available, we can define a more stringent age threshold or use age of onset as a quantitative trait in an AD population to distinguish these possibilities.

## Conclusions

In this study, we analyzed individuals who are APOE ε4 risk allele carriers but are resilient to Alzheimer’s disease, and discovered a novel AD protective genetic variants modifying AD risk in APOE ε4 risk allele carriers. Our analysis of Mount Sinai Biobank cohort led to the identification of a likely loss-of-function variant rs10553596 in CASP7 associated with significantly reduced AD incidence in elder populations. The protective effect of rs10553596 against AD was observed in four independent cohorts of European descent with similar odds ratios, and the overall effect in these cohorts was statistically significant. In addition, we show rs10553596 is associated in *cis* with lowered caspase 7 gene expression. Taken together, these results are consistent with the notion that caspase 7 plays a critical role in mediating microglial activation in CNS that triggers neurotoxicity and AD pathogenesis.

## Ethics approval and consent to participate

The study was approved by the Mount Sinai Institutional Review Board (IRB), and conducted in accordance with the Declaration of Helsinki and Good Clinical Practice (GCP). Informed consent was obtained from all participants in Mount Sinai Biobank cohort and Geisinger Health System (GHS) MyCode Cohort.

## Consent for publication

Not applicable.

## Availability of data and material

Not applicable.

## Additional file

Additional file 1: Figure S1. Relationship between the frequency of rs10553596 in CASP7 and APOE e4 allele frequency. (A) Variant frequencies across the 1000 genome phase 3 ethnic groups for rs10553596 and the APOE ε4 allele, The ethnic groups are color coded by continent/region. (B) The frequency of rs10553596 versus the APOE ε4 allele. **Figure S2.** Frequency bar chart showing the variant frequencies across all DIVAS disease and control cohorts for rs10553596. The y axis shows the allele frequencies. Blue bars represent healthy cohorts with different ethnicities. Red bars present diseased cohorts. FALS stands for familial Amyotrophic lateral sclerosis. (PDF 134 kb)
