# Supplementary material for: A loss of function variant in CASP7 protects against Alzheimer’s disease in homozygous APOE ε4 allele carriers
Source: BMC Genomics. 2016 Jun 23;17(Suppl 2):445. doi: 10.1186/s12864-016-2725-z (PMC4928152; doi:10.1186/s12864-016-2725-z)
Supplement: Additional file 1: Figure S1. — Relationship between the frequency of rs10553596 in CASP7 and APOE e4 allele frequency. (A) Variant frequencies across the 1000 genome phase 3 ethnic groups for rs10553596 and the APOE ε4 allele, The ethnic groups are color coded by continent/region. (B) The frequency of rs10553596 versus the APOE ε4 allele. Figure S2. Frequency bar chart showing the variant frequencies across all DIVAS disease and control cohorts for rs10553596. The y axis shows the allele frequencies. Blue bars represent healthy cohorts with different ethnicities. Red bars present diseased cohorts. FALS stands for familial Amyotrophic lateral sclerosis. (PDF 134 kb) [file 12864_2016_2725_MOESM1_ESM.pdf]

a)

POP	Population Description	Continent	Sample Size	CASP7 Freq	APOE ε4 Freq	APOE ε4 Hom Freq	APOE ε4 Het Freq
ESN	Esan in Nigeria	AFR	99	0.48	0.24	0.06	0.36
YRI	Yoruba in Ibadan, Nigeria	AFR	108	0.47	0.23	0.04	0.38
MSL	Mende in Sierra Leone	AFR	85	0.47	0.26	0.06	0.40
GWD	Gambian in Western Division, Gambia	AFR	113	0.45	0.27	0.08	0.39
ASW	African Ancestry in Southwest US	AFR	61	0.40	0.21	0.03	0.34
ACB	African Caribbean in Barbados	AFR	96	0.36	0.26	0.07	0.37
LWK	Luhya in Webuye, Kenya	AFR	99	0.34	0.38	0.15	0.46
BEB	Bengali in Bangladesh	SAS	86	0.32	0.09	0.01	0.15
PJL	Punjabi in Lahore,Pakistan	SAS	96	0.29	0.08	0.01	0.15
TSI	Toscani in Italia	EUR	107	0.29	0.10	-	0.21
PUR	Puerto Rican in Puerto Rico	AMR	104	0.28	0.11	-	0.21
STU	Sri Lankan Tamil in the UK	SAS	102	0.28	0.13	0.02	0.23
CHS	Southern Han Chinese	EAS	105	0.28	0.06	-	0.11
GBR	British in England and Scotland	EUR	91	0.27	0.18	0.04	0.26
KHV	Kinh in Ho Chi Minh City, Vietnam	EAS	99	0.26	0.09	0.02	0.14
JPT	Japanese in Tokyo	EAS	104	0.26	0.08	0.02	0.13
CDX	Chinese Dai in Xishuangbanna	EAS	93	0.25	0.10	-	0.20
GIH	Gujarati Indian in Houston,TX	SAS	103	0.25	0.05	0.01	0.08
FIN	Finnish in Finland	EUR	99	0.24	0.19	0.04	0.29
IBS	Iberian populations in Spain	EUR	107	0.22	0.14	0.01	0.26
ITU	Indian Telugu in the UK	SAS	102	0.21	0.08	-	0.17
CHB	Han Chinese in Bejing	EAS	103	0.21	0.10	-	0.20
CEU	Utah residents	EUR	99	0.19	0.18	0.02	0.31
CLM	Colombian in Medellin	AMR	94	0.17	0.15	0.02	0.27
MXL	Mexican Ancestry in Los Angeles, CA	AMR	64	0.16	0.09	-	0.17
PEL	Peruvian in Lima, Peru	AMR	85	0.15	0.06	-	0.12

b)

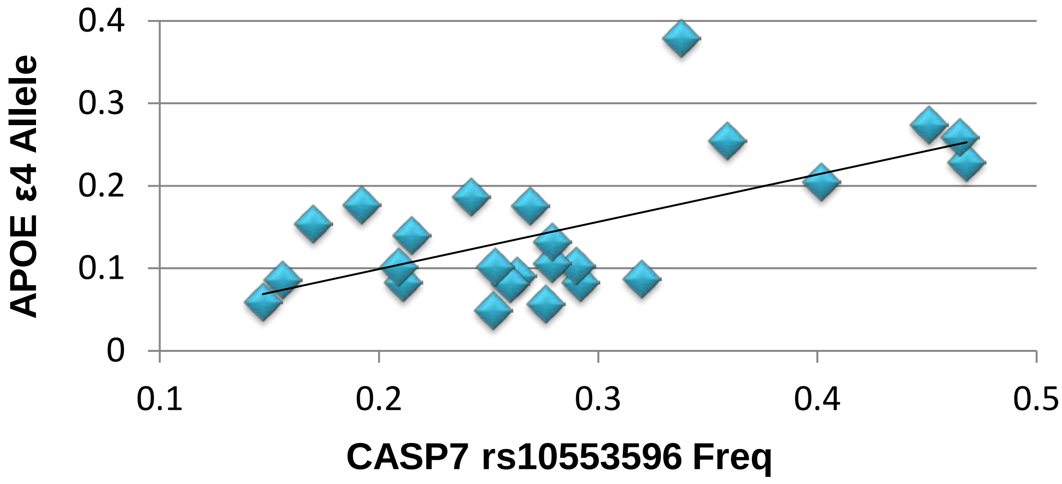

Figure S1: **The relationship between the frequency of rs10553596 in CASP7 and APOE ε4 allele frequency.** (a) Table showing the variant frequencies across the 1000g phase 3 ethnic groups for rs10553596 and the APOE e4 allele, The ethnic groups are color coded by continent/region. (b) Figure plotting the frequency of rs10553596 versus the APOE e4 allele.

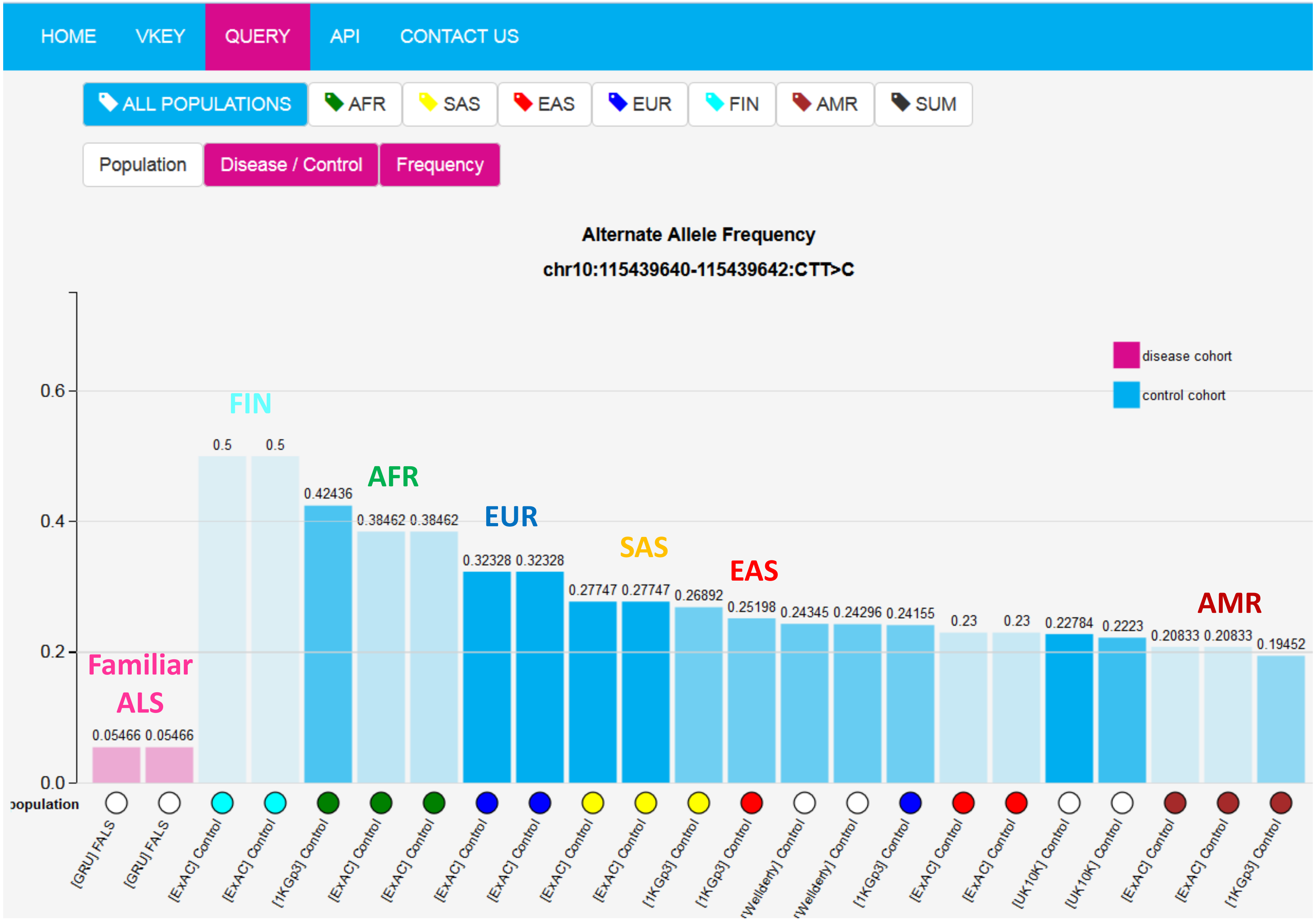

Figure S2: **Frequency bar chart showing the variant frequencies across all DIVAS disease and control cohorts for the CASP7 variant rs10553596**. Opacity of bars indicates the sample size of that cohort.
